# Comparisons of the Efficacy of Alpha Glucosidase Inhibitors on Type 2 Diabetes Patients between Asian and Caucasian

**DOI:** 10.1371/journal.pone.0079421

**Published:** 2013-11-13

**Authors:** Xiaoling Cai, Xueyao Han, Yingying Luo, Linong Ji

**Affiliations:** Endocrinology & Metabolism Department, Peking University People’s Hospital, Beijing, China; Univserity of Tolima, Colombia

## Abstract

**Background:**

To compare the efficacy of glycemic control and insulin secretion of alpha glucosidase inhibitors (AGI) on type 2 diabetes patients between Asian and Caucasian.

**Methodology/Principal Findings:**

The MEDLINE®, EMBASE®, CENTRAL were searched and qualified studies in Asian and Caucasian population comparing AGI treatment with placebo or other oral anti-diabetic drugs in type 2 diabetic patients were included. Totally 58 qualified studies were included. When AGI treatment was compared with placebo, a significant difference in HbA1c decline from baseline favoring AGI treatment was found in Asian (weighted mean difference (WMD), −0.50%; 95% CI, −0.66% to −0.34%) and in Caucasian a significant difference in HbA1c decline favoring AGI treatment was also found (WMD, −0.68%; 95% CI, −0.76% to −0.60%). In Asian, fasting plasma glucose was reduced with AGI treatment compared with placebo (WMD, −0.53 mmol/L; 95% CI, −0.91 to −0.14 mmol/L) and in Caucasian there was also a significant difference in FPG changes favoring AGI therapy (WMD, −0.88 mmol/L; 95% CI, −1.00 to −0.77 mmol/L). Studies in Asian showed a significant difference in fasting insulin changes favoring AGI treatment (WMD, −0.78 uU/ml; 95% CI, −0.96 to −0.59 uU/ml). While in Caucasian fasting insulin was decreased without significance with AGI treatment (WMD-1.24 uU/ml; 95% CI, −2.51 to 0.04 uU/ml). Body weight was decreased with AGI treatment in Asian (WMD, −1.00 kg; 95% CI, −1.69 to −0.31 kg) and was also decreased with AGI treatment in Caucasian (WMD, −0.73 kg; 95% CI, −1.13 to −0.33 kg).

**Conclusions/Significance:**

According to results from this meta-analysis, the efficacy in glucose lowering, body weight reduction and insulin secretion decreasing of AGI treatment in Asian were comparable with those in Caucasian.

## Introduction

In the treatment of type 2 diabetes, alpha-glucosidase inhibitors (AGIs; including acarbose, miglitol, voglibose) were recommend by guidelines for glucose control in type 2 diabetes. AGIs delay the absorption of carbohydrates by the gut, by inhibiting alpha-glucosidase in the small intestine, and thus have an effect on lowering postprandial blood glucose and insulin levels [1]–[3]. It was postulated that due to its mode of action, AGIs might be more efficacious in Asian population following an eastern diet with higher carbohydrate content than Caucasian population following a western diet [4], [5]. Although this is a reasonable assumption, it was not evidence based. Recently, there are some systemic reviews or meta-analysis evaluating the efficacy and safety of alpha-glucosidase inhibitors mainly in Caucasians [6], [7]. The present meta-analysis evaluated the clinical evidence of efficacy of AGIs in Asians and Caucasians and made a comparison of efficacy of AGIs between Asians and Caucasians.

## Methods

### Search Strategy

The following databases for primary studies during the stipulated period of time were searched: MEDLINE® (1966 to June 2012), EMBASE® (1974 to June 2012), the Cochrane Central Register of Controlled Trials (CENTRAL; 1966 to June 2012). The electronic search was first conducted in January 2012 and then repeated in June 2012. The main search concepts were type 2 diabetes, alpha-glucosidase inhibitors, acarbose, miglitol, voglibose, randomized controlled trials, Asian, Chinese, Japanese, Korean, Indian, etc. The PubMed strategy formed the basis for the strategies developed for the other electronic databases. We searched for additional trials in the prescribing information documents of approved medications, at relevant Web sites (http://www.clinicalstudyresults.org and http://www.clinicaltrials.gov).

### Study Selection

We defined anti-hyperglycemia efficacy of AGIs as placebo adjusted HbA1c changes from baseline after administration of AGIs treatment in placebo control in randomized clinical trials when we made a comparison of efficacy of AGIs between Asians and Caucasians. We also try to summarize the information on the relative efficacies of AGIs against other hypoglycemic agent by comparing the absolute reduction of HbA1c from baseline after administration of AGIs and other active oral hypoglycemia comparators in randomized clinical trials. Based on these analysis goal, we defined the inclusion criteria of studies as following: 1) placebo or active oral hyperglycemic agents controlled randomized controlled trials carried out in Asian countries as Asians; 2) placebo or active oral hyperglycemic agent controlled randomized controlled trials carried out in western countries as Caucasians; 2) The length of study was at least 12 weeks; 3) The index of glucose changes was change of HbA1c and fasting plasma glucose during the clinical trial from the baseline in the comparative groups. The contents of 441 abstracts or full-text manuscripts identified through the literature search were reviewed independently by two investigators (CXL, LYY) in duplicate to determine whether the study met eligibility criteria for inclusion. Where discrepancies between investigators occurred for inclusion or exclusion, a third investigator (HXY) was involved to conduct additional assessment of the study and discrepancies were resolved by discussion.

A validated 3-item scale was used to evaluate the overall reporting quality of the trials selected for inclusion in the present review. This scale provided scoring for randomization (0–2 points), double blinding (0–2 points), and withdrawals (1 point). Scores ranged from 0 to 5, and scores≥3 indicated a study of high quality [8].

### Data Abstraction

Similar to study selection, data abstraction was completed by two independent investigators (CXL, LYY). Discrepancies between the results of the abstraction were resolved by discussion and simultaneous reference to the relevant articles. Using a standardized form, the following data were collected: title, primary author’s name, year and source of publication, country of origin, study design, treatment allocation procedures, baseline characteristics of the study population (sample size, age, diabetic duration, HbA1c), description of the drug therapies, duration of the treatment, outcomes of diabetes (changes of HbA1c, fasting plasma glucose, fasting insulin, body weight, and hypoglycemic events). If data concerning the outcome were missing from an article, then the investigators attempted to contact the primary author in order to obtain the missing data.

### Statistical Analysis

This meta-analysis was conducted according to the PRISMA guidelines for the conduct and reporting of meta-analyses of RCTs [9]. Heterogeneity of the effect across studies was assessed by *Q* ^2^ statistics, which is distributed as χ^2^ statistics. A value of *P*<0.10 was used to indicate lack of homogeneity among effects. *I* ^2^ statistics were provided to quantify the percentage of total variation across studies that was attributable to heterogeneity rather than to chance. A value >50% represented substantial variability. A fixed-effect model was used when no significant heterogeneity was detected among subgroups. When significant heterogeneity was detected, its sources were first analyzed. In the absence of obvious clinical or other sources of heterogeneity, a random-effect model was used. We assessed publication bias by visual inspection of the funnel plot. All analyses were conducted in Review Manager, version 5.1. For continuous variables (HbA1c, fasting plasma glucose, weight, etc.), we calculated weighted mean differences (WMD) and 95% confidence intervals (CIs) for change from baseline in AGI vs comparator (placebo or active hypoglycemic agent) groups. Analyses were also made to test the subgroup differences between different ethnic groups. Randomized controlled trials carried out in Asian countries were defined as Asians, randomized controlled trials carried out in western countries were defined as Caucasians.

## Results

### Flow of Included Studies

**Figure 1:**
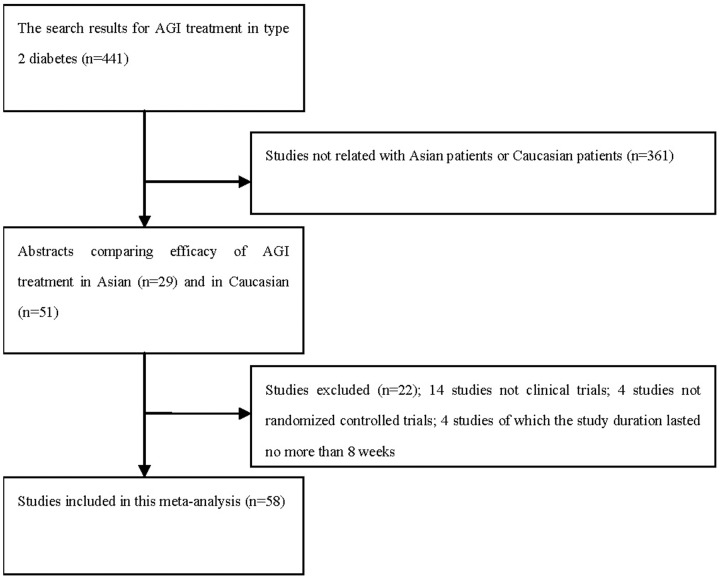
The flowchart of included studies.

From the search, after a review of the titles and abstracts, 441 abstracts were deemed eligible for further review, among which 29 studies were carried out in Asian population and 51 studies were in Caucasian population, and these studies were retrieved for more detailed evaluation. Then, 22 studies were excluded, in which, 14 studies were not clinical trials, 4 studies were not randomized controlled trials, and 4 studies lasted no more than 8 weeks. Figure 1 showed the flowchart of included studies in this analysis. According to the criteria of inclusion, a total of 58 studies were included in this meta-analysis. (Table S1 shows the characteristics of RCTs included.).

### Study Characteristics

There were totally 19 studies in Asian(13 for acarbose, 3 for voglibose, 3 for miglitol),in which 12 published trials comparing an AGI with placebo, given as monotherapy [10]–[13] or add-on therapy to other hypoglycemic agents [14]–[21], 8 published studies comparing an AGI with an active agents, given as monotherapy [12], [22]–[26] or add-on thearapy [27], [28]. There were totally 39 RCTs in Caucasians, in which 35 studies comparing an AGI with placebo (28 for acarbose, 7 for miglitol), given as monotherapy [29]–[48], [70], [71] or add-on thearapy [31], [42], [49]–[60], and 15studies comparing an AGI with an active agent (13 for acarbose, 2 for miglitol), given as monotherapy [32], [35], [37], [38], [46], [61]–[63], [70] or add-on therapy [57], [58], [64]–[68].

### Methodological Quality

All studies were randomized controlled trials including a control group (placebo or oral hypoglycemic agents). Eligibility criteria were clearly reported in all trials. The studies that were funded by pharmaceutical companies were clearly disclosed. Studies were restricted to randomized controlled trials to ensure the inclusion of only high-quality evidence.

### Changes in HbA1c for AGI Treatment vs Placebo or Active Agents in Asian

**Figure 2:**
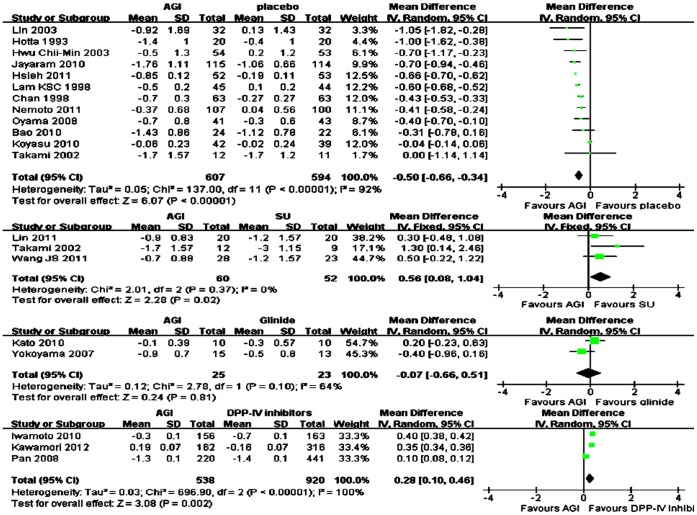
Weighted mean difference in change in HbA1c for AGI treatment versus placebo or active control in Asian.

Overall, AGI treatment (n = 607) was significantly associated with more reduction of mean HbA1c from baseline compared with placebo (n = 594) in Asians (WMD in change from baseline, −0.50%; 95% CI, −0.66% to −0.34%) (Figure 2). For monotherapy, compared with placebo (n = 133), AGI treatment (n = 137) was also significantly associated with more mean HbA1c change from baseline (WMD, −0.35%; 95% CI, −0.69% to 0.00%), and for add-on therapy, the reduction of mean HbA1c from baseline was also significantly more in AGI treatment (n = 470) compared with placebo (n = 461) (WMD, −0.59%; 95% CI, −0.67% to −0.50%). Among these studies, there were nine studies comparing arcabose with placebo, two studies comparing miglitol with placebo. Compared with placebo, arcabose treatment and miglitol treatment both were associated with more reduction of mean HbA1c from baseline (WMD for arcabose, −0.51%; 95% CI, −0.72% to −0.30%; WMD for miglitol, −0.64%; 95% CI, −0.69% to −0.60% respectively). Compared with sulfonylurea (SU) treatment (n = 52), AGI treatment (n = 60) was significantly associated with less reduction of mean HbA1c from baseline (WMD, 0.56%; 95% CI, 0.08% to 1.04%. Compared with DPP-IV inhibitors treatment (n = 920), AGI treatment (n = 538) was significantly associated with less reduction of mean HbA1c from baseline (WMD, 0.28%; 95% CI, 0.27% to 0.29%). Compared with glinide treatment (n = 23), the reduction of mean HbA1c from baseline was comparable with AGI treatment (n = 25) (WMD, −0.02%; 95% CI, −0.36% to 0.32%) (Figure 2).

### Changes in HbA1c for AGI Treatment vs Placebo or Active Agents in Caucasian

**Figure 3:**
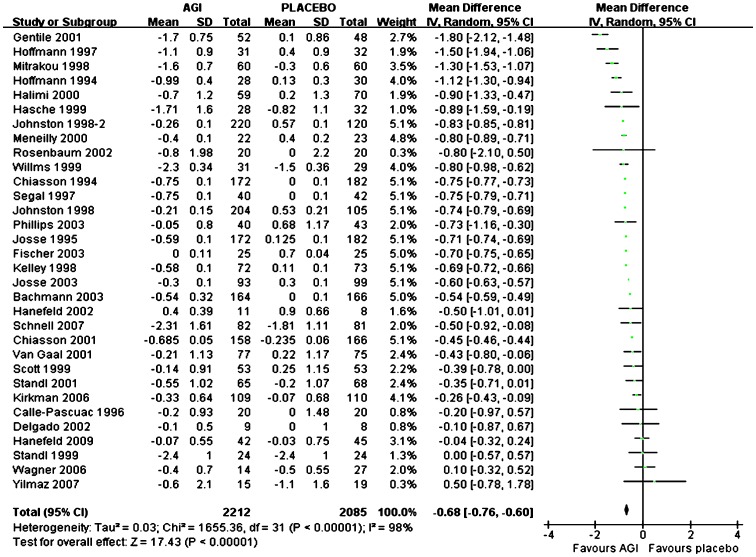
Weighted mean difference in change in HbA1c for AGI treatment versus placebo in Caucasian.

Overall, mean HbA1c reduction from baseline was significantly more with AGI treatment (n = 2212) compared with placebo (n = 2085) in Caucasians (WMD, −0.68%; 95% CI, −0.76% to −0.60%) (Figure 3). For monotherapy, compared with placebo (n = 1112), mean HbA1c reduction from baseline was significantly more with AGI treatment (n = 1275) (WMD, −0.68%; 95% CI, −0.77% to −0.58%), and for add-on therapy, mean HbA1c reduction from baseline was also significantly more with AGI treatment (n = 899) compared with placebo (n = 934) (WMD, −0.66%; 95% CI, −0.74% to −0.58%). Among these studies, there were twenty-five studies comparing arcabose with placebo, seven studies comparing miglitol with placebo. Compared with placebo, arcabose treatment and miglitol treatment both were associated with more reduction of mean HbA1c from baseline (WMD for arcabose, −0.68%; 95% CI, −0.74% to −0.61%; WMD for miglitol, −0.71%; 95% CI, −0.90% to −0.52% respectively). Compared with SUs treatment (n = 258), the reduction of mean HbA1c from baseline was comparable with AGI treatment (n = 259) (WMD, 0.29%; 95% CI, −0.37% to 0.95%). Compared with metformin treatment (n = 182), the reduction of mean HbA1c from baseline was comparable with AGI treatment (n = 168) (WMD, 0.35%; 95% CI, −0.15% to 0.86%). Compared with TZDs treatment (n = 336), AGI treatment (n = 326) was associated with significantly less reduction of mean HbA1c from baseline (WMD, 0.52%; 95% CI, 0.41% to 0.62%).

### Changes in Fasting Plasma Glucose (FPG) for AGI Treatment vs Placebo or Active Agents in Asian

**Figure 4:**
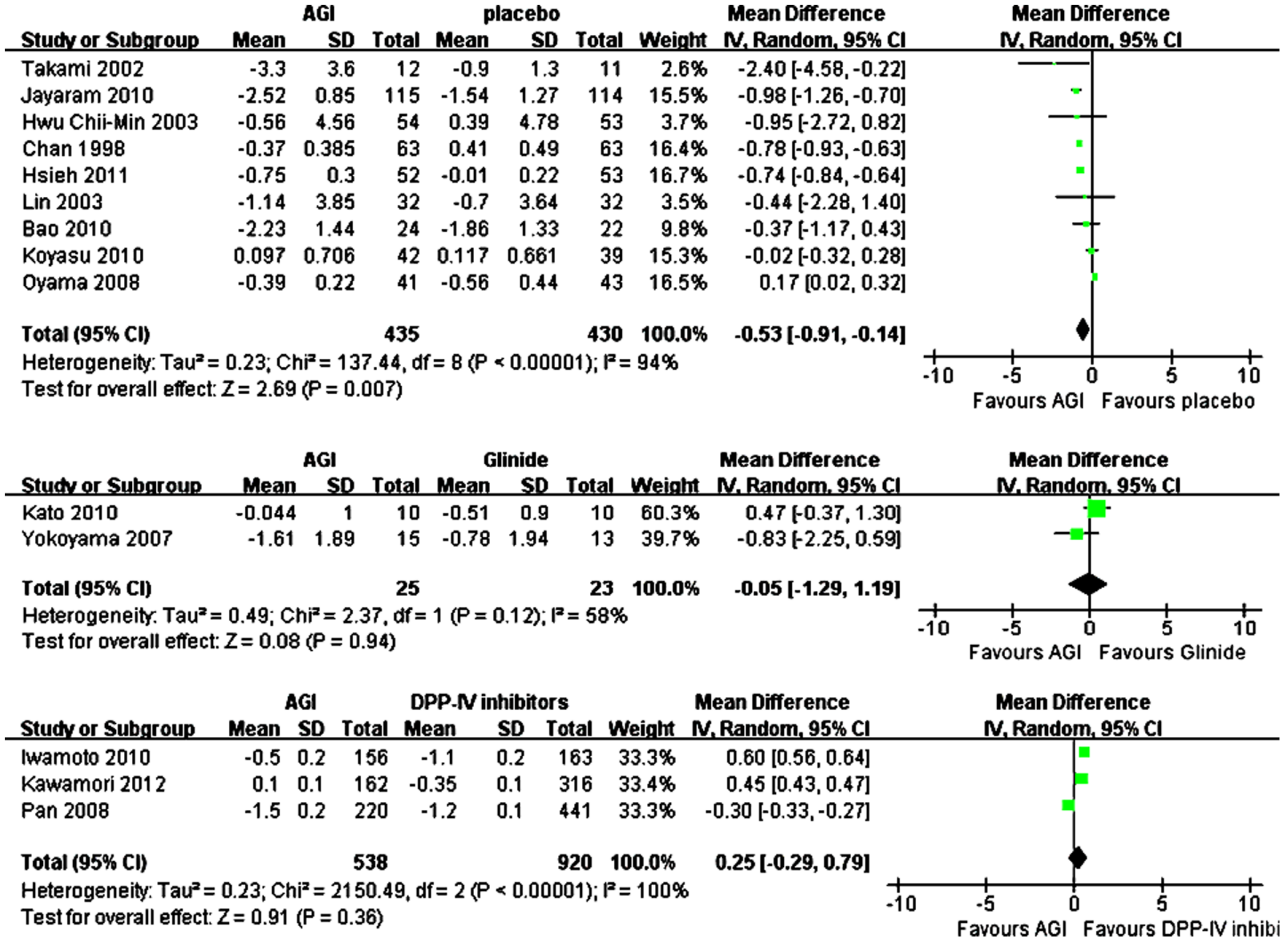
Weighted mean difference in changes in FPG for AGI treatment versus placebo or active control in Asian.

Overall, mean FPG reduction from baseline was significantly more with AGI treatment (n = 435) compared with placebo (n = 430) in Asians (WMD, −0.53 mmol/L; 95% CI, −0.91 to −0.14 mmol/L) (Figure 4). For monotherapy, compared with placebo (n = 113), mean FPG reduction from baseline was comparable with AGI treatment (n = 117) (WMD, −0.59 mmol/L; 95% CI, −1.32 to 0.14 mmol/L), and for add-on therapy, mean FPG reduction from baseline was also comparable with AGI treatment (n = 318) compared with placebo (n = 317) (WMD, −0.51 mmol/L; 95% CI, −1.07 to 0.04 mmol/L). Among these studies, there were seven studies comparing arcabose with placebo, and compared with placebo, arcabose treatment was associated with comparable reduction of mean FPG from baseline (WMD for arcabose, −0.43 mmol/L; 95% CI, −0.92 mmol/L to 0.07 mmol/L). Compared with DPP-IV inhibitors treatment (n = 920), AGI treatment (n = 538) was associated with comparable reduction of mean FPG from baseline (WMD, 0.25 mmol/L; 95% CI, −0.29 mmol/L to 0.79 mmol/L). Compared with glinide treatment (n = 23), the reduction of mean FPG from baseline was also comparable with AGI treatment (n = 25) (WMD, −0.05 mmol/L; 95% CI, −1.29 mmol/L to 1.19 mmol/L) (Figure 4).

### Changes in Fasting Plasma Glucose (FPG) for AGI Treatment vs Placebo or Active Agents in Caucasian

**Figure 5:**
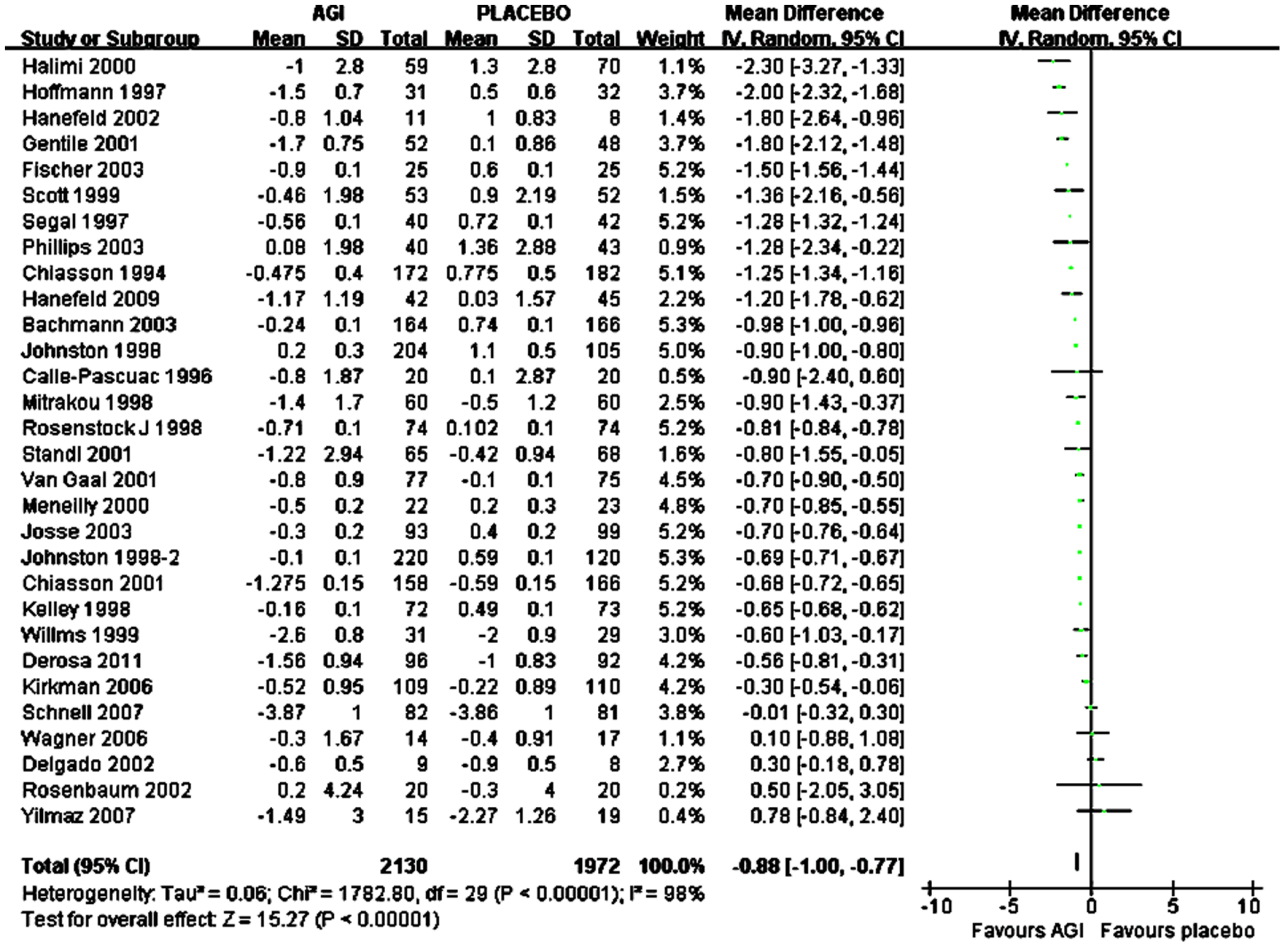
Weighted mean difference in changes in FPG for AGI treatment versus placebo in Caucasian.

Overall, mean FPG reduction from baseline was significantly more with AGI treatment (n = 2130) compared with placebo (n = 1972) in Caucasians (WMD, −0.88 mmol/L; 95% CI, −1.00 to −0.77 mmol/L) (Figure 5). For monotherapy, compared with placebo (n = 1026), mean FPG reduction from baseline was significantly more with AGI treatment (n = 1220) (WMD, −0.94 mmol/L; 95% CI, −1.18 to −0.71 mmol/L), and for add-on therapy, mean FPG reduction from baseline was also significantly more with AGI treatment (n = 949) compared with placebo (n = 984) (WMD, −0.84 mmol/L; 95% CI, −0.98 to −0.69 mmol/L). Among these studies, there were twenty-three studies comparing arcabose with placebo and seven studies comparing miglitol with placebo. Compared with placebo, arcabose treatment and miglitol treatment both were associated with more reduction of mean FPG from baseline (WMD for arcabose, −0.90 mmol/L; 95% CI, −1.04 mmol/L to −0.75 mmol/L; WMD for miglitol, −0.86 mmol/L; 95% CI, −1.08 mmol/L to −0.63 mmol/L respectively). Compared with SUs treatment (n = 231), the reduction of mean FPG from baseline was comparable with AGI treatment (n = 231) (WMD, 0.67 mmol/L; 95% CI, −0.65 mmol/L to 2.00 mmol/L). Compared with metformin treatment (n = 182), the reduction of mean FPG from baseline was comparable with AGI treatment (n = 168) (WMD, −0.15 mmol/L; 95% CI, −1.11 mmol/L to 0.81 mmol/L). Compared with TZDs treatment (n = 336), AGI treatment (n = 326) was associated with comparable reduction of mean FPG from baseline (WMD, 0.70 mmol/L; 95% CI, −0.41 mmol/L to 1.81 mmol/L).

### Changes in Fasting Insulin for AGI Treatment vs Placebo or Active Agents in Asian

Overall, mean fasting insulin reduction from baseline was significantly more with AGI treatment (n = 242) compared with placebo (n = 241) in Asians (WMD, −0.78 uU/ml; 95% CI, −0.96 to −0.59 uU/ml). For monotherapy, compared with placebo (n = 113), mean fasting insulin reduction from baseline was more with AGI treatment (n = 117) but without significance (WMD, −0.31 uU/ml; 95% CI, −1.13 to 0.51 uU/ml), and for add-on therapy, mean fasting insulin reduction from baseline was significantly more with AGI treatment (n = 125) compared with placebo (n = 128) (WMD, −0.80 uU/ml; 95% CI, −1.00 to −0.61 uU/ml). Among these studies, there were four studies comparing arcabose with placebo, and compared with placebo, arcabose treatment was associated with significantly more reduction of mean fasting insulin from baseline (WMD for arcabose, −0.78 uU/ml; 95% CI, −0.97 uU/ml to −0.59 uU/ml). Compared with DPP-IV inhibitors treatment (n = 479), AGI treatment (n = 318) was associated with significantly more reduction of mean fasting insulin from baseline (WMD, −0.54 uU/ml; 95% CI, −0.78 uU/ml to −0.30 uU/ml). Compared with glinide treatment (n = 23), the reduction of mean fasting insulin from baseline was comparable with AGI treatment (n = 25) (WMD, −0.65 uU/ml; 95% CI, −4.42 uU/ml to 3.12 uU/ml).

### Changes in Fasting Insulin for AGI Treatment vs Placebo or Active Agents in Caucasian

Overall, mean fasting insulin reduction from baseline was more with AGI treatment (n = 1293) compared with placebo (n = 1125) in Caucasians but without significance (WMD, −1.24 uU/ml; 95% CI, −2.51 to 0.04 uU/ml). For monotherapy, compared with placebo (n = 674), mean fasting insulin reduction from baseline was more with AGI treatment (n = 860) but without significance (WMD, −1.25 uU/ml; 95% CI, −2.71 to 0.20 uU/ml), and for add-on therapy, mean fasting insulin reduction from baseline was more with AGI treatment (n = 433) compared with placebo (n = 451) without significance (WMD, −1.11 uU/ml; 95% CI, −4.11 to 1.89 uU/ml). Among these studies, there were fifteen studies comparing arcabose with placebo and five studies comparing miglitol with placebo. Compared with placebo, arcabose treatment was associated with more reduction of mean fasting insulin from baseline (WMD for arcabose, −1.62 uU/ml; 95% CI, −3.21 uU/ml to −0.03 uU/ml), and miglitol treatment was associated with comparable reduction of mean fasting insulin from baseline (WMD for miglitol, −0.11 uU/ml; 95% CI, −1.20 uU/ml to 0.98 uU/ml). Compared with SUs treatment (n = 92), the reduction of mean fasting insulin from baseline was significantly more with AGI treatment (n = 91) (WMD, −0.38 uU/ml; 95% CI, −0.51 uU/ml to −0.26 uU/ml). Compared with metformin treatment (n = 123), the reduction of mean fasting insulin from baseline was comparable with AGI treatment (n = 122) (WMD, 1.99 uU/ml; 95% CI, −2.84 uU/ml to 6.81 uU/ml).

### Changes in Body Weight for AGI Treatment vs Placebo or Active Agents in Asian

Body weight reduction from baseline was significantly more with AGI treatment (n = 216) compared with placebo (n = 210) in Asians (WMD, −1.00 kg; 95% CI, −1.69 kg to −0.31 kg). Among these studies, there were four studies comparing arcabose with placebo, and compared with placebo, arcabose treatment was associated with significantly more reduction of mean weight from baseline (WMD for arcabose, −1.02 kg; 95% CI, −1.73 kg to −0.31 kg). Compared with DPP-IV inhibitors treatment (n = 604), AGI treatment (n = 376) was associated with significantly more reduction of mean weight from baseline (WMD, −1.00 kg; 95% CI, −1.59 kg to −0.40 kg). Compared with SUs treatment (n = 52), the reduction of mean weight from baseline was comparable with AGI treatment (n = 60) (WMD, −1.61 kg; 95% CI, −6.59 kg to 3.37 kg).

### Changes in Body Weight for AGI Treatment vs Placebo or Active Agents in Caucasian

In Caucasians, body weight reduction from baseline was significantly more with AGI treatment (n = 653) compared with placebo (n = 671) (WMD, −0.73 kg; 95% CI, −1.13 to −0.33 kg). Among these studies, there were ten studies comparing arcabose with placebo and four studies comparing miglitol with placebo. Compared with placebo, arcabose treatment was associated with comparable reduction of mean weight from baseline (WMD for arcabose, −0.33 kg; 95% CI, −0.67 kg to 0.01 kg), and miglitol treatment was associated with significantly more reduction of mean weight from baseline (WMD for miglitol, −1.17 kg; 95% CI, −1.63 kg to −0.7 kg). Compared with SUs treatment (n = 175), the reduction of mean weight from baseline was significantly more with AGI treatment (n = 173) (WMD, −2.77 kg; 95% CI, −3.3 kg to −2.24 kg). Compared with metformin treatment (n = 142), the reduction of mean weight from baseline was comparable with AGI treatment (n = 128) (WMD, −0.45 kg; 95% CI, −2.04 kg to 1.14 kg).

### Comparisons between Asian and Caucasian

Comparisons of HbA1c changes from baseline between Asian and Caucasian showed that when AGI was compared with placebo, the between-group difference was 0.08% without significance (95% CI, −0.21% to 0.38%, P>0.05), and when arcabose or miglitol was compared with placebo, the between-group difference was also without significance, respectively. Comparison of FPG changes between groups showed that when AGI was compared with placebo, the between-group difference was 0.31 mmol/L (95% CI, −0.22 to 0.84 mmol/L, P>0.05). When AGI was compared with placebo, the between-group difference of fasting insulin level was not significantly different (0.39 uU/ml (95% CI, −1.83 to 2.61 uU/ml, P>0.05), and the between-group difference of body weight was also comparable (0.51 kg (95% CI, −2.46 to 3.47 kg, P>0.05). Details were shown in Table 1.

**Table 1:** Comparisons between Asian and Caucasian in the WMD in changes of efficacy of AGI treatment versus placebo or active control treatment.

	Asian		Caucasian		Difference between two groups	
	WMD	95% CI	WMD	95% CI	Difference	95% CI
**HbA1c(%)**						
AGI vs placebo in monotherapy	−0.35	−0.69 to 0.00	−0.68	−0.77 to −0.58	0.28	−0.26 to 0.82
AGI vs placebo in add-on therapy	−0.59	−0.67 to −0.50	−0.66	−0.74 to −0.58	−0.06	−0.43 to 0.31
AGI vs placebo (total)	−0.50	−0.66 to −0.34	−0.68	−0.76 to −0.60	0.08	−0.21 to 0.38
Arcabose vs placebo	−0.51	−0.72 to −0.30	−0.68	−0.74 to −0.61	0.01	−0.35 to 0.37
Miglitol vs placebo	−0.64	−0.69 to −0.60	−0.71	−0.90 to −0.52	0.16	−0.43 to 0.74
AGI vs SU	0.56	0.08 to 1.04	0.29	−0.37 to 0.95	0.54	−1.01 to 2.10
AGI vs glinide	−0.02	−0.36 to 0.32	/	/	/	/
AGI vs DPP-IV inhibitors	0.28	0.27 to 0.29	/	/	/	/
AGI vs metformin	/	/	0.35	−0.15 to 0.86	/	/
AGI vs TZDs	/	/	0.52	0.41 to 0.62	/	/
**FPG(mmol/L)**						
AGI vs placebo in monotherapy	−0.59	−1.32 to 0.14	−0.94	−1.18 to −0.71	0.46	−0.66 to 1.58
AGI vs placebo in add-on therapy	−0.51	−1.07 to 0.04	−0.84	−0.98 to −0.69	0.23	−0.43 to 0.89
AGI vs placebo (total)	−0.53	−0.91 to −0.14	−0.88	−1.00 to −0.77	0.31	−0.22 to 0.84
Arcabose vs placebo	−0.43	−0.92 to 0.07	−0.90	−1.04 to −0.75	0.32	−0.31 to 0.99
Miglitol vs placebo	/	/	−0.86	−1.08 to −0.63	/	/
AGI vs SU	/	/	0.67	−0.65 to 2.00	/	/
AGI vs glinide	−0.05	−1.29 to 1.19	/	/	/	/
AGI vs DPP-IV inhibitors	0.25	−0.29 to 0.79	/	/	/	/
AGI vs metformin	/	/	−0.15	−1.11 to 0.81	/	/
AGI vs TZDs	/	/	0.70	−0.41 to 1.81	/	/
**Fasting insulin (uU/ml)**						
AGI vs placebo in monotherapy	−0.31	−1.13 to 0.51	−1.25	−2.71 to 0.20	1.40	−2.01 to 4.81
AGI vs placebo in add-on therapy	−0.80	−1.00 to −0.61	−1.11	−4.11 to 1.89	−0.70	−4.01 to 2.61
AGI vs placebo (total)	−0.78	−0.96 to −0.59	−1.24	−2.51 to 0.04	0.39	−1.83 to 2.61
Arcabose vs placebo	−0.78	−0.97 to −0.59	−1.62	−3.21 to −0.03	0.13	−2.78 to 3.04
Miglitol vs placebo	/	/	−0.11	−1.20 to 0.98	/	/
AGI vs SU	/	/	−0.38	−0.50 to −0.26	/	/
AGI vs glinide	−0.65	−4.42 to 3.12	/	/	/	/
AGI vs DPP-IV inhibitors	−0.54	−0.78 to −0.30	/	/	/	/
AGI vs metformin	/	/	1.99	−2.84 to 6.81	/	/
**Weight(kg)**						
AGI vs placebo (total)	−1.00	−1.69 to −0.31	−0.73	−1.13 to −0.33	0.51	−2.46 to 3.47
Arcabose vs placebo	−1.02	−1.73 to −0.31	−0.33	−0.67 to 0.01	0.37	−3.51 to 4.24
Miglitol vs placebo	/	/	−1.17	−1.63 to −0.70	/	/
AGI vs SU	−1.61	−6.59 to 3.37	−2.77	−3.30 to −2.24	1.13	−1.75 to 4.02
AGI vs DPP-IV inhibitors	−1.00	−1.59 to −0.40	/	/	/	/
AGI vs metformin	/	/	−0.45	−2.04 to 1.14	/	/

## Discussion

In this meta-analysis, we had analysis the effect of AGIs on glucose, insulin secretion and weight against placebo or other active oral hypoglycemic agents in Asian and Caucasian type 2 diabetes patients. The analysis suggested that treatment with AGIs (acarbose, voglibose and miglitol) lead to comparable changes of HbA1c and body weight in type 2 diabetes patients in Asian and Caucasian population when compared with placebo and other active oral hypoglycemic agents. In this analysis, we also observed a comparable change of insulin level between Asian and Caucasian population associated with AGIs treatment.

In terms of clinical efficacy, as measured by the reduction in HbA1c and FPG from baseline after adjusting placebo effect, across trials of 12–52 weeks, AGI treatment produced a mean HbA1c reduction difference of 0.50% and a mean FPG reduction difference of 0.53 mmol/L, respectively, in comparison with placebo in Asian. But compared with active agents (mainly sulphonylurea), HbA1c was reduced not favoring AGI treatment. In Caucasians, AGI treatment produced comparable HbA1c and FPG reduction from baseline (0.68% and 0.88 mmol/L respectively) after adjusting placebo effect. The results of our meta-analysis are in accordance with some results concluded mainly in Caucasians previously. As one meta-analysis reported by Van de Laar [6] indicated that in clinical trials (36 trials in Caucasians and 5 trials in Asians), after adjusting placebo effect, acarbose decreased HbA1c by 0.77% (95% CI 0.64–0.90%) and miglitol by 0.68% (95% CI 0.44–0.93%), voglibose, yielded a difference of 0.47% in favor of voglibose (95% CI 0.31–0.63%). For FPG, after adjusting for placebo effects, acarbose treatment is associated with a mean FPG reduction of 1.09 mmol/l (28 comparisons; 95% CI 0.83–1.36), miglitol 0.52 mmol/l (2 comparisons; 95% CI 0.16–0.88), and voglibose 0.60 mmol/l (1 comparison; 95% CI 0.23–0.97). And the overall comparison of acarbose with sulfonylurea yielded a non-significant HbA1c reduction of 0.38% (95% CI −0.02–0.77%) favoring sulfonylurea treatment. Derosa [7] in a systemic review (15 trials in Caucasians and 4 trials in Asians) concluded that treatment with acarbose was more effective than placebo in improving HbA1c levels and in reducing FPG levels after 7 months of therapy, but did not give the exact weighted mean difference.

In terms of insulin secretion, compared with placebo or active agents, treatment with AGI in Asian showed a more reduction in fasting insulin from baseline of 0.78 uU/ml and 0.55 uU/ml respectively. While in Caucasians, AGI treatment showed a decrease in fasting insulin of 1.24 uU/ml when compared with placebo and a trend of increase when compared with active agents. However, there was no significant difference between Asian and Caucasian in fasting insulin level changes in response to AGI treatment. Van de Laar [6] reported that compared with placebo, acarbose had no effect on fasting insulin levels and a lowering effect on 1-h postload insulin levels of 40.8 pmol/l (95% CI 21.0–50.6pmol/l) mainly in Caucasians. For systemic review in Asian patients, no comparison is available.

In terms of weight changes, treatment of AGI produced a weight reduction of 1.0 kg either when compared with placebo or active agents in Asian. In Caucasians, AGI treatment produced a weight decrease of 0.73 kg and 1.79 kg respectively compared with placebo and active agents. Weight changes between Asian and Caucasian after adjusting placebo effect were also comparable. However, what we found seems to be different from previous studies. Van de Laar [6] found in a meta analysis concluded mainly in Caucasians (36 trials in Caucasians and 5 trials in Asians) that acarbose had a statistically significant decreasing effect on BMI of 0.17 kg/m^2^, but the effect on the outcome “body weight” was not statistically significant.

In dis-concordance with the result of our meta analysis, A recent meta-analysis [69] had shown that acarbose achieved a greater absolute reduction of HbA1c levels in the Eastern diet type 2 diabetes population than in the Western diet type 2 diabetes population. Based on this observation, the author suggested that AGIs are more efficacious in type 2 diabetes of eastern population. Although this was an interesting observation, we had noticed that qualities of some studies in Eastern diet group in this article were low level and should not be included in the meta-analysis for reason of publication bias and performance bias.

As a meta-analysis, we should admit that there are several potential limitations. The glycemic control of the AGI group and the control group was not optimal in several studies. The included studies used different targets for HbA1c or FPG to guide the titration of hypoglycemic agents. The including criteria and the baseline characteristics of selected studies were different. Most of the trials were not long term, generally lasting less than 1 year, and few evaluated important clinical outcomes, such as cardiovascular events and death. Reporting bias may also be a concern. Whatever, we pooled the results of a group of trials with the aim of evaluating the efficacy and other non-glycemic effects of AGI treatment in Asian, and drawing comparisons between Asian and Caucasians of these effects.

This article may be the first research that made a whole systemic review of AGI treatment in Asian and also the first research that made comparisons of efficacy of AGI treatment between Asian and Caucasian. The observation made from this study might provide evidence for guideline development and clinical treatment. According to this meta-analysis, what we have found is that the efficacy in glucose lowering, body weight reduction and insulin secretion decreasing of AGI treatment in Asian is comparable with that in Caucasian.

## Supporting Information

Table S1 **Characteristics of Randomized Controlled Trials of Alpha glucosidase Inhibitors Included in the Systematic Review.**
(DOCX)

Checklist S1 **A PRISMA checklist for this meta-analysis.**
(DOC)
