# Supplementary material for: Comparisons of the Efficacy of Alpha Glucosidase Inhibitors on Type 2 Diabetes Patients between Asian and Caucasian
Source: PLoS One. 2013 Nov 13;8(11):e79421. doi: 10.1371/journal.pone.0079421 (PMC3827349; doi:10.1371/journal.pone.0079421)
Supplement: Table S1 — Characteristics of Randomized Controlled Trials of Alpha glucosidase Inhibitors Included in the Systematic Review. (DOCX) [file pone.0079421.s001.docx]

**Supplement table**

Table supplement: Characteristics of Randomized Controlled Trials of Alpha glucosidase Inhibitors Included in the Systematic Review*.

| Author , year | Study duration | Treatment group | Number of patients | Age (years) | Men (%) | DM duration(months) | HbA1c (%) |  |
| --- | --- | --- | --- | --- | --- | --- | --- | --- |
| **Asians** | | | | | | | |  |
| Placebo compared mono therapy | | | | | | | |  |
| CHAN 1998(10) | 24 weeks | AGI | 63 | 52.8 (10.2) | 50.8 | 32.4±42 | -0.7(-1,-0.39) |  |
|  |  | Placebo | 63 | 54 (10) | 50.8 | 25.2±40.8 | -0.27(-0.54,0) |  |
| HOTTA 1993(11) | 24 weeks | AGI | 20 | 49.8 | 26.3 | 55.2 | 11.1 |  |
|  |  | Placebo | 20 | 47.9 | 22.2 | 57.6 | 10.3 |  |
| Takami, 2002(12) | 3 months | voglibose | 12 | / | 25.00 | Newly diagnosed | 8±1.7 |  |
|  |  | Placebo | 11 | / | 36.36 | Newly diagnosed | 8.3±1.3 |  |
|  |  | glyburide | 9 | / | 33.33 | Newly diagnosed | 9.1±2.5 |  |
| Koyasu, 2010(13) | 1 year | AGI | 42 | 66.1±8.6 | 90.5 | Newly diagnosed | 5.55±0.38 |  |
|  |  | Placebo | 39 | 66.5±8 | 92.3 | Newly diagnosed | 5.59±0.39 |  |
| Placebo compared add-on therapy(or combination therapy) | | | | | | | |  |
| Jayaram S, 2010(14) | 12 weeks | AGI+met | 115 | 49.33±7.7 | 69.6 | / | 9.47±0.6 |  |
|  |  | Placebo+met | 114 | 49.01±8.45 | 71.1 | / | 9.32±0.65 |  |
| BAO YQ, 2010(15) | 8 weeks | AGI+Glipizide | 22 | 52.6 | / | Newly diagnosed | 8.01±0.87 |  |
|  |  | Placebo+Glipizide | 24 | 54.7 | / | Newly diagnosed | 7.88±0.79 |  |
| Hwu Chii-Min, 2003(16) | 18 weeks | AGI | 54 | 58.1±8.4 | / | 160.7±109.3 | 9.5±0.8 |  |
|  |  | Placebo | 53 | 54.7±8.6 | / | 131±73.6 | 9.5±1 |  |
| Lin BJ, 2003(17) | 24 weeks | AGI | 32 | 57.7±7.3 | 53.1 | 7 | 9.01±1.2 |  |
|  |  | Placebo | 32 | 55.4±8.5 | 37.5 | 5 | 8.99±0.95 |  |
| Lam KSL,1998(18) | 24 weeks | AGI | 45 | 57.8±1.3 | 44.4 | 122±8.4 | 9.5±0.1 |  |
|  |  | Placebo | 44 | 56.9±1.3 | 43.2 | 121.5±9.9 | 9.4±0.1 |  |
| Oyama 2008(19) | 12 months | Placebo | 43 | 63±4 | 41.86 | 11.3±5.2 | 7.4±0.7 |  |
|  |  | AGI | 41 | 65±6 | 29.27 | 11.7±6.5 | 7.5±0.8 |  |
| Hsieh 2011(20) | 24 weeks | Miglitol+SU | 52 | 58.4±10.5 | 79.3 | / | 8.14±0.72 |  |
|  |  | Placebo+SU | 53 | 59±10.7 | 51.4 | / | 8.11±0.77 |  |
| Nemoto 2011(21) | 12 weeks | Miglitol+insulin | 107 | / | / | / | / |  |
|  |  | Placebo+insulin | 100 | / | / | / | / |  |
| Active agents compared mono therapy | | | | | | | |  |
| Kato, 2010(22) | 12 weeks | AGI | 10 | 67.6±6.2 | 70 | Newly diagnosed | 6±0.3 |  |
|  |  | nateglinide | 10 | 67.8±8.6 | 70 | Newly diagnosed | 6.1±0.6 |  |
| Takami, 2002 | 3 months | voglibose | 12 | / | 25.00 | Newly diagnosed | 8±1.7 |  |
|  |  | Placebo | 11 | / | 36.36 | Newly diagnosed | 8.3±1.3 |  |
|  |  | glyburide | 9 | / | 33.33 | Newly diagnosed | 9.1±2.5 |  |
| Yokoyama 2007(23) | 3 months | Miglitol | 15 | 57±9 | 86.6 | 4±4 | 7.4±0.8 |  |
|  |  | Mitiglinide | 13 | 60±10 | 76.9 | 3±3 | 7.4±0.6 |  |
| Pan, 2008(25) | 24 weeks | vildagliptin | 441 | 51.8 ± 10.1 | 60.1 | 1.2 ± 2.4 | 8.6 ± 0.9 |  |
|  |  | acarbose | 220 | 51.9 ± 10.3 | 63.2 | 1.3 ± 2.4 | 8.6 ± 1.0 |  |
| Iwamoto,2010(26) | 12 weeks | Sitagliptin | 163 | 60.8 (10.1) | 72.4 | 5.1 (4.8) | 7.8 (0.9) |  |
|  |  | voglibose | 156 | 60.6 (10.0) | 60.3 | 5.3 (4.9) | 7.8 (0.9) |  |
| Kawamori,2012(24) | 26 weeks | Linagliptin 5 mg | 159 | 60.3 (9.4) | 69.8 | / | 8.07 (0.66) |  |
|  |  | Linagliptin 10 mg | 160 | 61.3 (10.0) | 70.0 | / | 7.98 (0.68) |  |
|  |  | voglibose | 162 | 58.5 (9.9) | 71.0 | / | 8.02 (0.71) |  |
| Active agents compared add-on therapy | | | | | | | |  |
| WANG JS, 2011(27) | 24 weeks | AGI | 28 | 52.8±8.2 | 53.6 | 7.6 | 8.2±0.8 |  |
|  |  | glibenclamide | 23 | 54.7±8.3 | 43.5 | 6 | 8.6±1.6 |  |
| LIN SD, 2011(28) | 24 weeks | AGI+met | 20 | 51.5±8 | 65 | 7±4.8 | 8.7±1.6 |  |
|  |  | Glibenclamide+met | 20 | 53.9±8.5 | 50 | 5.7±4.2 | 8.2±0.8 |  |
| **Caucasians** | | | | | | | |  |
| Placebo compared mono therapy | | | | | | | |  |
| Calle-Pascuac, 1996(29) | 16 weeks | AGI | 20 | / | / | / | 6.3±0.8 |  |
|  |  | Placebo | 20 | / | / | / | 6.4±1.3 |  |
| Derosa, 2011(30) | 7 months | AGI | 96 | / | 49.0 | / | 6.8±0.6 |  |
|  |  | Placebo | 92 | / | 48.9 | / | 6.7±0.5 |  |
| Chiasson, 1994(31) | 1 year | AGI | 38 | / | / | / | / |  |
|  |  | Placebo | 39 | / | / | / | / |  |
| Chiasson, 2001(32) | 36 weeks | Placebo | 83 | 57.7±9.9 | 67.5 | 5.1±4.9 years | 8.1±0.7 |  |
|  |  | Miglitol | 82 | 57.3±9 | 78.0 | 5.2±4.7 years | 8.2±0.9 |  |
| Delgado,2011(33) | 4 months | AGI | 9 | / | / | / | 6.8±0.5 |  |
|  |  | Placebo | 8 | / | / | / | 7.5±0.6 |  |
| Gentile, 2001(34) | 28 weeks | AGI | 52 | / | / | / | 8.9±0.8 |  |
|  |  | Placebo | 48 | / | / | / | 8.7±0.9 |  |
| Hanefeld,2002(35) | 16 weeks | AGI | 11 | 60.4±1.3 | 91 | 92.4±16.2 | 8.2±0.3 |  |
|  |  | Placebo | 8 | 59±1.6 | 75 | 81.2±19.2 | 8.7±0.6 |  |
| Hanefeld,2009(36) | 20 weeks | AGI | 42 | 62.33±8.7 | / | / | 6.11±0.48 |  |
|  |  | Placebo | 45 | 59.92±10.05 | / |  | 6.09±0.66 |  |
| Hoffmann,1997(37) | 24 weeks | AGI | 31 | 58.9±9.4 | 19 | 36.9±27.2 | 9.6±0.9 |  |
|  |  | Placebo | 32 | 60.2±8.6 | 38 | 43.2±33.9 | 9.4±0.9 |  |
| Hoffmann,1994(38) | 24 weeks | AGI | 28 | 58.8±6.9 | 46 | 12.7±10.8 | 8.29±0.42 |  |
|  |  | Placebo | 30 | 56.9±6.7 | 40 | 12.1±10.8 | 8.29±0.37 |  |
| Kirkman,2006(39) | 5 years | AGI | 109 | 53.7±11 | 33 | / | 6.35±0.65 |  |
|  |  | Placebo | 110 | 53.7±11.7 | 34.6 | / | 6.33±0.63 |  |
| Meneilly,2000(40) | 12 months | AGI | 22 | 68±1 | / | / | 7.3±0.1 |  |
|  |  | Placebo | 23 | 70±1 | / | / | 7±0.2 |  |
| Rosenbaum,2002(41) | 22 weeks | AGI | 20 | 59.8±8.2 | 30 | 82(6-240) | 6.4±1.7 |  |
|  |  | Placebo | 20 | 62±9.7 | 40 | 81(6-174) | 6.3±2.1 |  |
| Josse,1995(42) | 12 months | AGI | 38 | 57.2±1.1 | / | 5.2±0.6 | / |  |
|  |  | Placebo | 39 | / | / | / | / |  |
| Josse,2003(43) | 12 months | AGI | 93 | 69.7±0.5 | / | 5.8±0.7 | 7.4±0.1 |  |
|  |  | Placebo | 99 | 70.3±0.5 | / | 4.8±0.5 | 7.3±0.1 |  |
| Scott,1999(44) | 16 weeks | AGI | 53 | 56±9 | 62 | 21±15 | 7.0±0.87 |  |
|  |  | Placebo | 52 | 57±8 | 65 | 26±17 | 6.89±0.85 |  |
| Wagner,2006(45) | 12 weeks | AGI | 14 | 57(52-58) | 57.1 | 3.5(1-6) | 5.9(5.1-6.6) |  |
|  |  | Placebo | 17 | 54(50-58) | 82.3 | 4(2-5) | 6.6(6.1-7.1) |  |
| Segal,1997(46) | 24 weeks | Miglitol | 40 | 61 | 55 | / | 7.95 |  |
|  |  | Placebo | 42 | 59 | 57.1 | / | 8.25 |  |
| Johnston,1998(47) | 1 year | Miglitol | 204 | 55±1 | 50 | 5.1±0.5 | 8.67±0.14 |  |
|  |  | Placebo | 105 | 56.9±1.3 | 51 | 4.5±0.7 | 8.62±0.18 |  |
| Johnston,1998-2(48) | 1 year | Miglitol | 220 | 52.9 | / | 5.7 | 8.7 |  |
|  |  | Placebo | 120 | 53.9 | / | 4.8 | 8.53 |  |
| Fischer, 2003(70) | 16 weeks | AGI | 25 | 59.4±5.6 | / | 94±11.9 | 8.1±0.2 |  |
|  |  | Placebo | 25 | 58.6±6.3 | / | 77.3±10.7 | 8.3±0.2 |  |
| Hasche, 1999(71) | 24 months | AGI | 52 | / | / | / | 8.9±0.8 |  |
|  |  | Placebo | 48 | / | / | / | 8.7±0.9 |  |
| Placebo compared add-on therapy(or combination therapy) | | | | | | | |  |
| Bachmann, 2003(49) | 78 weeks | AGI+SU | 164 | 63.8±7.1 | 47.6 | 96±160 | 9.42±0.66 |  |
|  |  | Placebo+SU | 166 | 63.3±7.2 | 43.3 | 96±150 | 9.38±0.73 |  |
| Chiasson, 1994 | 1 year | AGI+MET | 83 | / | / | / | / |  |
|  |  | Placebo+MET | / | / | / | / | / |  |
|  |  | AGI+SU | 103 | / | / | / | / |  |
|  |  | Placebo+SU | / | / | / | / | / |  |
|  |  | AGI+insulin | 91 | / | / | / | / |  |
|  |  | Placebo+insulin | / | / | / | / | / |  |
| Chiasson, 2001 | 36 weeks | Placebo+MET | 83 | 57.9±8.6 | 73.5 | 7.5±7.4 years | 8.2±0.9 |  |
|  |  | Miglitol+MET | 76 | 58.9±7.9 | 77.6 | 6.1±5.5 years | 8.3±0.8 |  |
| Halimi,2000(50) | 6 months | AGI+MET | 59 | 56±9.2 | 47.4 | 114±89 | 8.6±1.1 |  |
|  |  | Placebo+MET | 70 | 55±10 | 62.8 | 108±90 | 8.5±1.1 |  |
| Kelley,1998(51) | 24 weeks | AGI+insulin | 72 | 61.8 | 63 | 12.5 | 8.77 |  |
|  |  | Placebo+insulin | 73 | 60.8 | 48 | 12.3 | 8.69 |  |
| Phillips,2003(52) | 24 weeks | AGI+MET | 40 | 58.37±10.7 | 65 | 5.32±4.55 | 8.05±0.89 |  |
|  |  | Placebo+MET | 43 | 62.39±8.02 | 76.7 | 6.06±5.32 | 7.82±0.83 |  |
| Rosenstock,1998(53) | 24 weeks | AGI+MET | 74 | 57.2 | 61 | 7.2 | 8.46 |  |
|  |  | Placebo+MET | 74 | 55.9 | 49 | 7.8 | 8.17 |  |
| Josse,1995 | 12 months | AGI+MET | 41 | 57.4±1.1 | 63.8 | 8.8±0.6 | / |  |
|  |  | Placebo+MET | 42 | / | / | / | / |  |
|  |  | AGI+SU | 52 | 58.4±0.9 | 56.3 | 9.4±0.7 | / |  |
|  |  | Placebo+SU | 51 | / | / | / | / |  |
|  |  | AGI+insulin | 41 | 56.6±0.9 | 57.1 | 12.9±0.8 | / |  |
|  |  | Placebo+insulin | 50 | / | / | / | / |  |
| Schnell,2007(54) | 20 weeks | AGI+insulin | 82 | 61.5±8.9 | / | 11.5±7 | 9.8±1.5 |  |
|  |  | Placebo+insulin | 81 | 62.3±7.4 | / | 9.6±5.1 | 9.4±1 |  |
| Standl,1999(55) | 24 weeks | AGI+insulin | 24 | 59.3±8.5 | / | 137.7±81.7 | 10.9±1 |  |
|  |  | Placebo+insulin | 24 | 62.9±9.4 | / | 146.7±68 | 11.0±1.2 |  |
| Standl,2001(56) | 24 weeks | Miglitol+SU+MET | 65 | 62±8 | 50.8 | 96(36-321) | 8.83±0.85 |  |
|  |  | Placebo+ SU+MET | 68 | 61±8 | 54.4 | 108(30-384) | 8.84±0.66 |  |
| Willms,1999(57) | 12 weeks | AGI+SU | 31 | 60.3±8.8 | 48.4 | 134.8±81.8 | 10.6±1.3 |  |
|  |  | Placebo+ SU | 29 | 59.2±9.4 | 58.6 | 119.6±76.5 | 10.6±1.6 |  |
| Yilmaz,2007(58) | 6 months | AGI+insulin | 15 | 62.6±6.6 | 46.7 | 13.9±7.2 | 8.3±2 |  |
|  |  | Placebo+insulin | 19 | 61.5±12 | 36.8 | 17.9±11.5 | 8.7±1.6 |  |
| Van Gaal,2001(59) | 32 weeks | Miglitol+MET | 77 | 57.9±10 | 41.5 | 6(1-32) | 8.5±1 |  |
|  |  | Placebo+ MET | 75 | 57.9±8.5 | 49.3 | 6(0-22) | 8.4±1 |  |
| Mitrakou.1998(60) | 24 weeks | Miglitol+insulin | 60 | 57.4±5.6 | 48.3 | 101.8±53.7 | 9.9±0.5 |  |
|  |  | Placebo+ insulin | 60 | 57.4±5.8 | 61.7 | 94.4±38.8 | 9.9±0.4 |  |
| Active agents compared mono therapy | | | | | | | |  |
| Chiasson, 2001 | 36 weeks | Miglitol | 82 | 57.3±9 | 78.0 | 5.2±4.7 years | 8.2±0.9 |  |
|  |  | metformin | 83 | 57.9±8.6 | 73.5 | 7.5±7.4 years | 8.2±0.9 |  |
| Feinböck,2003(61) | 20 weeks | AGI | 108 | / | / | / | / |  |
|  |  | Glimepiride | 111 | / | / | / | / |  |
| Göke,2002(62) | 26 weeks | AGI | 136 | 58.8±9.1 | 54.5 | 59.1±50.3 | 9.03±1.32 |  |
|  |  | Pioglitazone | 129 | 58.9±9.1 | 53.5 | 57±55.4 | 8.98±1.2 |  |
| Hanefeld,2002 | 16 weeks | AGI | 11 | 60.4±1.3 | 90.9 | 92.4±16.2 | 8.2±0.3 |  |
|  |  | Glibenclamide | 8 | 60.6±2.5 | 62.5 | 85.4±14.7 | 8.4±0.4 |  |
| Hoffmann,1997 | 24 weeks | AGI | 31 | 58.9±9.4 | 19 | 36.9±27.2 | 9.6±0.9 |  |
|  |  | metformin | 31 | 55.9±7.8 | 45 | 25±17.4 | 9.7±0.9 |  |
| Hoffmann,1994 | 24 weeks | AGI | 28 | 58.8±6.9 | 46 | 12.7±10.8 | 8.29±0.42 |  |
|  |  | Glibenclamide | 27 | 59.5±5.7 | 48 | 17.6±13.1 | 8.3±0.37 |  |
| Salman,2001(63) | 24 weeks | AGI | 27 | 52.6±9.1 | / | 4.2±3.4 | 8.9±0.7 |  |
|  |  | Gliclazide | 30 | 56.1±8.7 | / | 4.7±5.6 | 8.7±0.6 |  |
| Segal,1997 | 24 weeks | Miglitol | 40 | 61 | 55 | / | 7.95 |  |
|  |  | Glibenclamide | 37 | 56 | 62.2 | / | 7.96 |  |
| Fischer, 2003 | 16 weeks | AGI | 25 | 59.4±5.6 | / | 94±11.9 | 8.1±0.2 |  |
|  |  | Glibenclamide | 27 | 58.1±7 | / | 69.5±9.6 | 8.3±0.2 |  |
| Active agents compared add-on therapy | | | | | | | |  |
| Bayraktar,1996(64) | 8 weeks | AGI+SU | / | / | / | / | 10.6±0.6 |  |
|  |  | metformin+SU | / | / | / | / | 10.3±0.4 |  |
| Derosa,2009,2010(65,66) | 9 months | AGI+SU+MET | 175 | 57±6 | 49.7 | / | 8.0±0.6 |  |
|  |  | Pioglitazone+SU+MET | 175 | 55±8 | 49.1 | / | 7.9±0.5 |  |
| Duran, 2009(67) | 13 weeks | Repaglinide+glargine | 20 | 53.5±5.9 | 60 | / | 10.9±1.4 |  |
|  |  | AGI+glargine | 18 | 55.1±7.2 | 44.4 | / | 11±1.4 |  |
| Güvener,1999(68) | 6 months | AGI+insulin | 20 | 59.61±2.07 | 20 | 10.15±1.75 | 8.6±0.15 |  |
|  |  | Gliclazide+insulin | 18 | 53.1±1.37 | 22.2 | 11.86±1.56 | 8.32±0.26 |  |
| Willms,1999 | 12 weeks | AGI+SU | 31 | 60.3±8.8 | 48.4 | 134.8±81.8 | 10.6±1.3 |  |
|  |  | metformin+SU | 27 | 53.4±8.2 | 48.1 | 111.9±82.4 | 10.6±1.4 |  |
| Yilmaz,2007 | 6 months | AGI+insulin | 15 | 62.6±6.6 | 46.7 | 13.9±7.2 | 8.3±2 |  |
|  |  | metformin+insulin | 17 | 57.7±8.5 | 35.3 | 12.1±7.7 | 8.9±1.2 |  |
|  |  | Rosiglitazone+insulin | 15 | 57.6±8.8 | 53.3 | 12.1±7.9 | 9.6±1 |  |

*: No significant difference of baseline demographics was found between the Asian group and Caucasian group.
